# Headache-related clinical features in teleworkers and their association with coping strategies during the COVID-19 pandemic

**DOI:** 10.3389/fpubh.2023.1303394

**Published:** 2023-12-28

**Authors:** Mariève Houle, Julien Ducas, Arnaud Lardon, Martin Descarreaux, Andrée-Anne Marchand, Jacques Abboud

**Affiliations:** ^1^Department of Anatomy, Université du Québec à Trois-Rivières, Trois-Rivières, QC, Canada; ^2^Groupe de recherche sur les affections neuromusculosquelettiques (GRAN), Trois-Rivières, QC, Canada; ^3^Department of Human Kinetics, Université du Québec à Trois-Rivières, Trois-Rivières, QC, Canada; ^4^Institut Franco-Européen de Chiropraxie, Paris, France; ^5^Department of Chiropractic, Université du Québec à Trois-Rivières, Trois-Rivières, QC, Canada

**Keywords:** teleworkers, working from home, primary headache, coping strategies, COVID-19

## Abstract

**Objectives:**

The objectives were (1) to describe and compare headache-related clinical features between teleworkers with migraine and those with tension-type headache (TTH) and (2) to determine the association between coping strategies and headache frequency, and intensity in the context of the COVID-19 pandemic.

**Methods:**

This cross-sectional online survey was conducted with 284 teleworkers (127 with migraine and 157 with TTH). Sociodemographic data, information related to work factors, headache clinical features, coping strategies used during the COVID-19 pandemic, and headache-related clinical features were compared between headache profiles. Bivariate logistic regression analyses were used to determine the association between coping strategies and headache frequency, and intensity.

**Results:**

Results showed that teleworkers with migraine had longer and more painful headache episodes than teleworkers with TTH (*ps* < 0.001). Higher migraine frequency was associated with the use of the denial coping strategy (*p* = 0.006) while lower migraine intensity was associated with planning (*p* = 0.046) and the use of positive reframing (*p* = 0.025). Higher TTH frequency was associated with the use of venting, self-blame, and behavioral disengagement (*ps* < 0.007) while higher TTH intensity was associated with substance use and behavioral disengagement (*p*s < 0.030). All associations remained significant after adjusting for BMI as a covariate.

**Discussion/conclusion:**

Teleworkers with migraine had more intense and longer headache episodes than teleworkers with TTH. This could be explained by the fact that a greater proportion of individuals suffering from migraine experienced headaches prior to the beginning of the pandemic compared with teleworkers suffering from TTH. Regarding coping strategies, both primary headache profiles were associated with different types of coping strategies. Most of the coping strategies associated with headache frequency or intensity were maladaptive except for planning and positive reframing that were found to be inversely associated with migraine intensity.

## Introduction

The COVID-19 pandemic caused by the coronavirus named SARS-CoV-2 has significantly affected the economy and the business model (e.g., supply chain, consumer demands, sales and marketing) of almost all countries and territories (1). In fact, the COVID-19 pandemic has tremendously challenged organizations and companies around the globe and caused important changes related to the management of employees especially in industrialized countries (2). Drastic changes were implemented to respect government recommendations such as social distancing in the workplace (3–5) with the objective of reducing the infection rate (6–8). Such recommendations forced organizations and companies worldwide to rapidly implement teleworking regardless of their past experiences and work environment (5, 9).

Teleworking consists of an alternative arrangement allowing employees to work outside of the employer’s premises with the support of information and communication technologies (ICTs) such as laptops, smartphones, and tablets (9, 10). Traditionally, teleworking allows individuals to work either at home or at other offices and shared facilities (11, 12). However, since the beginning of the COVID-19 pandemic, the term telework has been more often used to define home-based telework (13). A survey conducted in 2020 in the United States reported that in 50% of the companies, more than 80% of human resources employees were working from home during the early stage of the COVID-19 pandemic (13). In Canada, the prevalence of home-based telework from all working domains has drastically increased from 5% in 2018 to almost 45% in April 2020 (14). In early 2021, while work restrictions related to the COVID-19 were eased off, teleworkers still accounted for one third of all workers (14).

Now that teleworking is well implemented in several working environments, it seems relevant to investigate health-related conditions that are often reported by teleworkers. Knowing that primary headaches such as migraine and tension-type headache (TTH) are considered one of the leading causes of disability in the general population, especially among people under 50 years of age (15), it is important to investigate if teleworkers present a similar headache-clinical profile to headache sufferers in the general population and how headache-related clinical features could be influenced by a stressful situation such as a pandemic. In the general population, the prevalence of migraine and TTH are, respectively, 11 and 42% (16). In a recent study, a large proportion of teleworkers (61%) reported having at least one headache episode during a typical work week (17). It is important to point out that primary headache profiles are influenced by non-modifiable and modifiable factors. Non-modifiable risk factors include age and sex, while modifiable risk factors include physical factors such as BMI and psychological factors such as stress, anxiety and medication overuse (18, 19). Thus, headache self-management during a new stressful situation such as the COVID-19 pandemic which has been identified as a collective trauma (20, 21) could represent an important challenge for teleworkers living with primary headaches.

When facing a stressful event or situation such as a pandemic, a large range of coping strategies can be used to improve people’s quality of life (22). Coping strategies involve a dynamic process that consists of a series of actions or responses based on how the individual and the environment interact together as well as how they influence each other. In addition, these actions or responses include cognitive, emotional, behavioral and physiological domains (23). People can use more than one coping strategy when attempting to decrease the physical, emotional and psychological burdens associated with stressful life situations such as a pandemic (24). One important thing is that when considering situational coping strategies, actions (what a person did) or responses (how a person will react) are related to a specific situation (episode or period of time) (25). Situational coping strategies can be dichotomised in different ways. Some authors divide coping strategies into problem-focused (efforts to control or change a specific stressor) and emotion-focused (efforts to manage the emotional response to a specific stressor) while others divide situational coping strategies into positive coping strategies (e.g., seeking social support and humor) or maladaptive coping strategies (e.g., alcohol consumption and self-blame) (22, 26). Maladaptive coping strategies have been recognized to immediately reduce the stress associated with a particular condition, but are also known to have negative long-term consequences on quality of life (27). In headache populations, problem-focused coping strategies have been found to be effective in the management of headache-related clinical features as well as in the management of the stressful situation itself. In fact, people using problem-focused strategies are more likely to find solutions to manage their condition (28, 29). Previous studies showed that both positive and maladaptive coping strategies were used by people when facing an infectious disease outbreak (30, 31). Knowing that maladaptive coping strategies are associated with pain in many health-related conditions including headache (32) and that a large proportion of teleworkers previously reported having headache episodes (17), it is important to address the impact of coping strategies on headache-related clinical features. The COVID-19 pandemic represents a unique multifaceted “stress circumstance” offering a distinctive opportunity to study coping strategies in teleworkers with primary headaches.

The first aim of this study was to describe and compare the clinical features of migraine and TTH episodes in teleworkers in the context of the COVID-19 pandemic. The second aim was to determine the association between coping strategies and headache frequency, and intensity in teleworkers with migraine or TTH in the context of the COVID-19 pandemic. We hypothesized that headache-related clinical features in teleworkers would be similar to what is found in the general population, meaning that teleworkers with migraine would report higher headache intensity and headache-related limitations than teleworkers with TTH while headache frequency would be lower in teleworkers with migraine than in those with TTH. We also hypothesized that maladaptive coping strategies would be associated with higher headache frequency, intensity, and limitations in both headache types.

## Methods

### Study design

This study is an observational cross-sectional study. Recruitment and data collection were conducted online from June 2021 to February 2022.

### Participants

Four hundred and one participants were recruited via social media platforms (Facebook pages and UQTR institutional web platform). To be included, participants needed to be aged between 18 and 75 years old, have had at least one headache episode (TTH or migraine) unrelated to COVID-19 infection or COVID-19 vaccination in the last 12 months, have been in a telework situation since the beginning of the pandemic (March, 14th, 2020) for at least 3 days per week and be able to understand and express themselves in French. Participants who were in a telework situation before March 2020 were excluded from this study. The project received approval from the Human Research Ethics Board of Université du Québec à Trois-Rivières (CER-21-277-07.18) and all methods were performed in accordance with the relevant guidelines and regulations. All participants provided informed written consent before completing the online survey.

### Data collection

Participants were asked to complete an online survey (Qualtrics, Provo, Utah, United States). The online survey included three sections: (1) sociodemographic data and information related to work factors, (2) headache-related clinical features, and (3) use of coping strategies.

#### Sociodemographic data and information related to work factors

In the first section of the survey, participants were asked to report their height, weight, age, and gender. To identify the specific waves of the COVID-19 pandemic, the completion date of the online survey was noted. More precisely, the COVID-19 context was identified using the COVID-19 timeline of the Institut National de Santé Publique du Québec (INSPQ). According to the INSPQ, surveys completed between March 21st and July 17th, 2021 were considered in the third wave, surveys completed between July 18th and December 4th, 2021 were considered in the fourth wave and surveys completed between December 5th and the closing time of the online survey (March 25th, 2022) were considered in the fifth wave.

Participants were asked to report any recent change in their type of work, to indicate their mean working hours during a typical work week and to specify how many of these were spent in a telework situation (mean working hours in a telework situation). Participants were then asked to indicate if they had a designated office space at home.

#### Headache-related clinical features

Headache-related clinical features such as headache status, presence or not of headaches before the beginning of the pandemic (March 14th, 2020), frequency, duration and intensity were assessed in the second section of the survey with open-ended questions. Then, participants were invited to indicate how their headache status had changed since the beginning of the pandemic using a 7-point Likert scale (1 = strongly improved, 4 = no changes, 7 = strongly deteriorated). In addition, mean headache episode duration and mean pain intensity since the beginning of the pandemic as well as during the last month, were questioned. Headache frequency during the last month was also questioned. Headache frequency and mean headache episode duration were assessed using open-ended questions while intensity was assessed using a 11-point numeric rating scale (NRS) (0 = no pain, 10 = worst pain imaginable) (33). Participants were also invited to indicate if they were using medication more than 50% of the time to manage their headache. In addition, participants were asked to indicate how their capacity to accomplish daily living activities had changed since the pandemic using a 7-point Likert scale (1 = strongly improved, 4 = no changes, 7 = strongly deteriorated). Participants also had to indicate how frequently their headaches had limited their activities of daily living during the last month using a 5-point Likert scale (1 = never, 3 = sometimes, 5 = always).

#### Coping strategies

The third section of the online survey aimed to assess teleworkers’ coping strategies in the context of the COVID-19 pandemic. Coping strategies were assessed using the French validated version of the Brief-COPE questionnaire (34). For each of the 28 items, participants were asked to identify on a 4-point Likert scale ranging from “I have not been doing this at all” (score = 1) to “I have been doing this a lot” (score = 4) how they react to the COVID-19 pandemic (35).

### Variable definitions

Individual headache profile (migraine or TTH) was determined using a series of statements drawn from the International Headache Society (IHS) 3rd edition International Classification of Headache Disorders criteria (36). Two general unlabeled profiles were presented to participants, one corresponding to migraine and the other corresponding to TTH. As the profiles were unlabeled, participants had to choose the profile that corresponded best to their symptoms instead of choosing the diagnosis they thought they had. Participants were asked to select which profile corresponded best to their symptoms. Then, all headache symptoms pertaining to migraine and TTH were presented, and participants were asked to select all symptoms that were associated with their headache episodes. During data extraction, a health care professional (chiropractor) verified the correspondence between the profile chosen by the participants and the symptoms they reported. If there was a discordance between the profile and the reported symptoms, the participant was excluded from the study. However, no distinction between migraine with and without aura was made.

#### Primary objective

For the first objective, headache profile (migraine or TTH) was identified as the independent variable. All other variables were considered as dependent variables. Change in headache status and change in capacity during activities of daily living were classified into 3 categories based on the 7-point Likert scale used in the survey. A score between 1 and 3 indicated an improvement, a score of 4 indicated that there was no change while a score between 5 and 7 indicated a deterioration of the condition.

The Brief-COPE questionnaire was used to assess situational coping strategies and responses in relation to the COVID-19 pandemic. This questionnaire includes 28 questions that are paired to obtain 14 different categories of coping strategies (active coping, planning, use of instrumental support, use of emotional support, venting, positive reframing, acceptance, denial, self-blame, humor, religion, self-distraction, substance use and behavioral disengagement) (35). The score for each of the 14 categories was obtained by adding up the score of the two items related to a given category from the original 28-item questionnaire. Each of the 14 different categories of coping strategy has a maximum possible score of 8 points, with each item within the category being worth a maximum of 4 points (35).

#### Secondary objective

For the second objective, monthly headache frequency and intensity were considered for the analyses. Headache frequency was divided into headache subcategories according to the IHS classification which provides 2 categories of headache frequency for migraine (episodic migraine = less than 15 episodes per month and chronic migraine = more than 15 episodes per month) and 3 categories for TTH (infrequent episodic TTH = less than 1 episode per month (or less than 12 episodes per year), frequent episodic TTH = between 1 and 14 episodes per month, and chronic TTH = at least 15 episodes per month) (36). Headache intensity for both migraine and TTH profiles was divided into 3 categories (mild = 0–4, moderate = 5–6 and severe = 7–10) (37). All variable definitions are presented in Table 1.

**Table 1:** Independent and dependent variable definitions.

Objective 1. Comparison between headache profiles	
Independent variables	Definition
Headache profile	Migraine | (2) TTH
Dependent variables	Definitions
Sociodemographic data	
Height	Height (m)
Weight	Weight (Kg)
BMI	Body mass index (Kg/m^2^)
Age	Age (years)
Gender	M: F: O
COVID-19 waves	3rd wave (March 21st – July 17th, 2021) 4th wave (July 18th – December 4th, 2021) 5th wave (December 5th, 2021) - end of the data collection (March 25th, 2022)
Work related information	
Work change	Changes related to the type of work (yes or no question)
Mean working hours per week	Mean of working hours during a typical work week (hours)
Mean telework hours per week	Mean of working hours in a telework situation during a typical work week (hours)
Designated office space	Presence of a designated workplace at home for telework (yes or no question)
Headache-related clinical features	
Headache status	Having headaches before March 14th, 2020 (yes or no question)
Change in headache status	Changes related to the headache status since the beginning of the pandemic (7-point Likert scale)
Monthly headache frequency	Number of headache episodes during the last month
Mean headache episode duration since the beginning of the pandemic	Mean headache duration since the beginning of the pandemic (minutes)
Mean headache episode duration during the last month	Mean headache duration during the last month (minutes)
Mean headache intensity since the beginning of the pandemic	Mean headache intensity since the beginning of the pandemic (/10)
Monthly headache intensity	Mean headache intensity during the last month (/10)
Medication intake since the beginning of the pandemic	Use of any medication more than 50% of the time to decrease the duration of a headache episode since the beginning of the pandemic (yes or no question)
Medication intake during the last month	Use of any medication more than 50% of the time to decrease the duration of a headache episode during the last month (yes or no question)
Change in capacity during activities of daily living	Changes in the capacity to perform daily activities when having an episode of headache since the beginning of the pandemic (5-point Likert scale)
Headache-related limitations during activities of daily living	Frequency of headache episodes that impact the capacity to perform daily activities during the last month (5-point Likert scale)
Coping strategies	
Brief-COPE	Response of participants when facing a stressful event (COVID-19 pandemic)
Objective 2. Associations between coping strategies and headache frequency, and intensity	
Independent variable	Definition
Brief-COPE	Response of participants when facing a stressful event (COVID-19 pandemic)
Dependent variables	Definitions
Headache frequency	Categories of headache frequency based on the frequency reported during the last month and following the IHS classification Migraine: episodic (<15 episodes per month), chronic (≥15 episodes per month) TTH: infrequent episodic (<1 episode per month) (less than 12 per years), frequent episodic (1–14 episodes per month), chronic (≥15 episodes per month)
Headache intensity	Categories of headache intensity (mild = 0–4, moderate = 5–6, severe = 7–8) based on the mean intensity reported during the last month

### Statistical analysis

For both objectives, if a participant did not provide an answer to 3 or more questions on the online survey or did not provide an answer to one of the questions related to coping strategies, he or she was excluded from the analysis. Any data whose value was greater than 3 times the standard deviation above the mean was considered aberrant and was removed from the related analysis (38). The large sample size allowed the use of parametric tests such as t-test and regression analysis.

To answer the first objective, t-tests for independent groups and chi-square tests were conducted to compare sociodemographic data, working status, headache clinical features and coping strategies. T-tests were used for continuous data (mean and standard deviation) while chi-square tests were used for dichotomous data (proportion). To answer the second objective, bivariate or ordinal logistic regression analyses were used to determine the association between each of the 14 coping strategies and headaches (migraine or TTH) frequency, and intensity. Associations were therefore carried out one by one and those that proved to be significant were adjusted for gender, age and BMI. Analyses were performed using the Statistical Package for the Social Sciences (SPSS) version 28 software (IBM, USA) and STATA.12® (StataCorp, Texas, USA). The level of significance was set at value of *p* ≤0.05.

## Results

### Description of teleworkers and comparison between headache profiles

**Figure 1:**
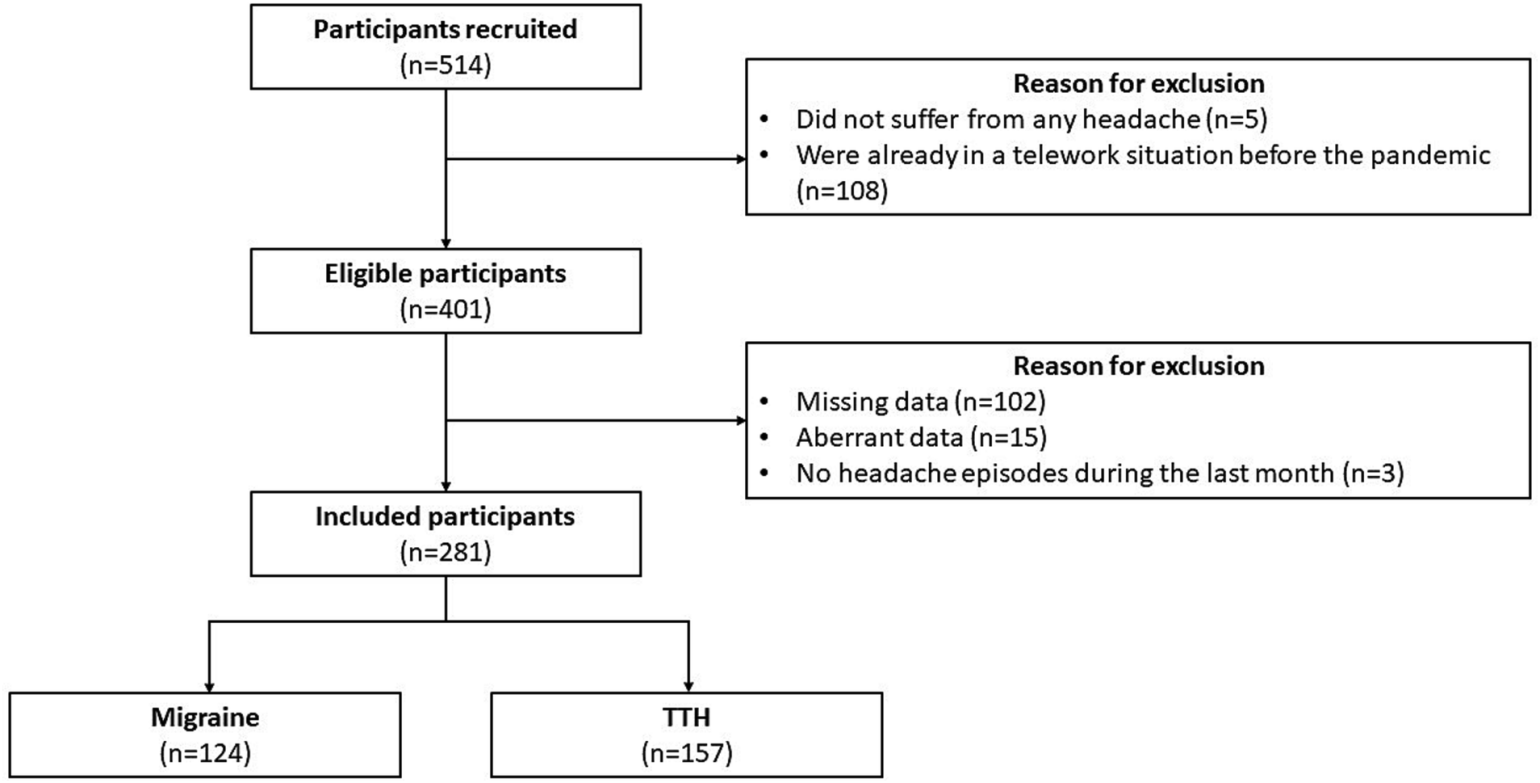
Participants flowchart.

Out of 514 potential participants recruited, 401 fulfilled all inclusion criteria. One hundred twenty participants were excluded based on aberrant data, incomplete questionnaires or because they had no headache episodes during the last month, leaving a total of 281 teleworkers included in this study (Figure 1).

Following the headache profile analysis based on the criteria from the IHS, 124 teleworkers had a headache profile corresponding to migraine (110 with episodic migraine, 14 with chronic migraine) while 157 had a headache profile corresponding to TTH (33 with infrequent TTH, 102 with frequent TTH and 22 with chronic TTH). Results from the t-tests for independent groups and chi-square analysis for sociodemographic data, work factors and headache clinical features are presented in Table 2. There was no significant difference in sociodemographic data and work-related variables between teleworkers with migraine and those with TTH (*ps* = [0.199–0.761]). Regarding headache clinical features, 78.23% of teleworkers with migraine had experienced headache episodes before the pandemic while this proportion was significantly lower in teleworkers with TTH (54.78%; *p* < 0.001). In addition, teleworkers with migraine reported having longer and more painful headache episodes (*ps* < 0.001), and using medication more often since the beginning of the pandemic (*p* = 0.047) and during the last month (*p* = 0.012). Only monthly headache frequency was considered similar between teleworkers with migraine and those with TTH (*p* = 0.072).

**Table 2:** Comparison of the sociodemographic data, work factors and headache-related clinical features between headache profiles.

Variables	Migraine profile (*n* = 124) Mean ± SD	TTH profile (*n* = 157) Mean ± SD	*T*-test value of *p*	Chi-square value of *p*
**Sociodemographic data**				
Height (m)	1.66 ± 0.10	1.66 ± 0.09	0.761	–
Weight (kg)	74.74 ± 19.55	73.68 ± 20.07	0.659	–
BMI (kg/m^2^)	26.67 ± 6.68	26.67 ± 6.68	0.584	–
Age (years)	37.61 ± 8.89	38.18 ± 9.68	0.611	–
Gender (M: F: O)	9: 115: 0	10: 146: 1	–	0.647
COVID-19 waves (1: 2: 3)	16: 40: 68	27: 44: 86	–	0.535
**Information related to work factors**				
Mean working hours per week (hours)	35.26 ± 6.66	36.38 ± 7.08	0.179	–
Mean telework hours per week (hours)	33.55 ± 7.73	34.49 ± 7.35	0.302	–
Designated office space (Yes % / No %)	86.29 / 13.71	84.71 / 15.29	–	0.710
**Headache clinical features**				
Headache status (Yes % / No %)	78.23 / 21.77	54.78 / 45.22	–	< 0.001
Monthly headache frequency	6.28 ± 6.40	6.68 ± 9.98	0.072	–
Mean headache episode duration since the beginning of the pandemic (min)	618.99 ± 970.41	249.32 ± 444.53	< 0.001	–
Mean headache episode duration during the last month (min)	561.36 ± 848.90	230.99 ± 423.76	< 0.001	–
Mean headache episode intensity since the beginning of the pandemic (/10)	6.63 ± 1.30	5.47 ± 1.56	< 0.001	–
Monthly headache intensity (/10)	6.45 ± 1.77	5.34 ± 1.92	< 0.001	–
Medication intake since the beginning of the pandemic (Yes % / No %)	78.23 / 21.77	67.52 / 32.48	–	0.047
Medication intake during the last month (Yes % / No %)	80.65 / 19.35	67.31 / 32.69	–	0.012

**Figure 2:**
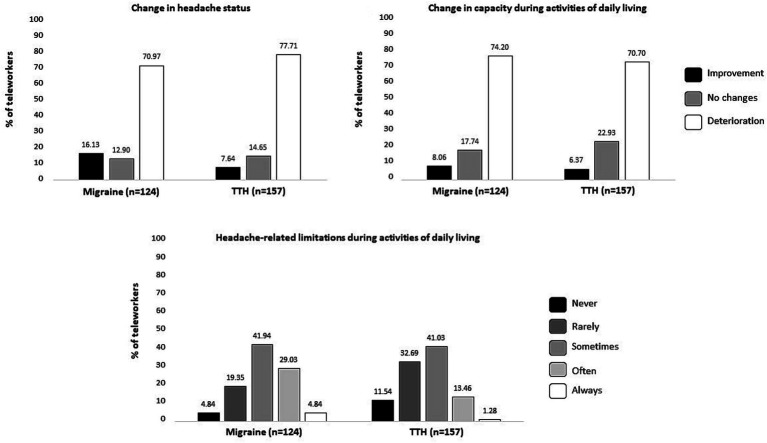
Change in headache status, change in capacity to perform activities of daily living as well as headache-related limitations during activities of daily living based on teleworkers headache profiles.

The results of the two measures related to change (i.e., change in headache status and change in capacity to perform activities of daily living) as well as the frequency of headache-related limitations during activities of daily living are illustrated in Figure 2. Results from the chi-square analyses showed that the proportion of teleworkers reporting a change in their headache status or a change in their capacity to perform activities of daily living since the beginning of the pandemic was similar between migraine and TTH profiles (*ps* = [0.084–0.522]). However, teleworkers with migraine reported more frequent headache-related limitations (*p* < 0.001) than teleworkers with TTH.

**Figure 3:**
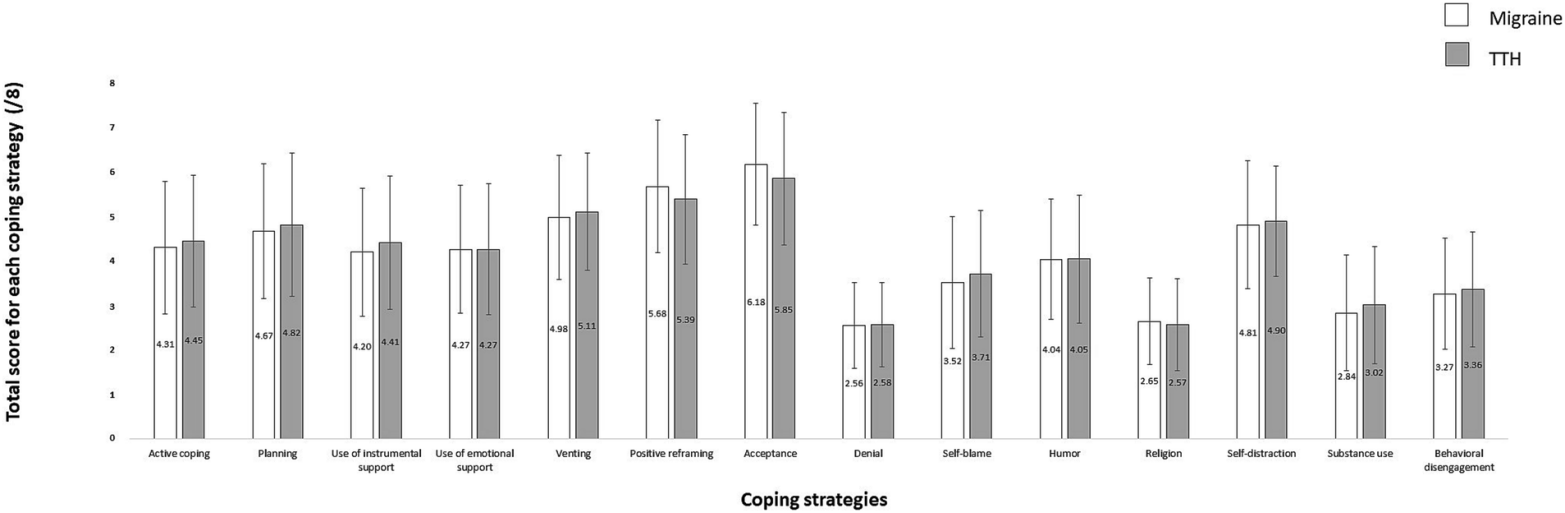
Comparison of utilization rate for each of the 14 coping strategies between headache profiles.

Regarding coping strategies, teleworkers with migraine reported using between 6 and 14 coping strategies while teleworkers with TTH reported using between 2 and 14 coping strategies to face the COVID-19 pandemic. Comparison of each coping strategy between headache profiles showed that teleworkers with migraine used all coping strategies as frequently as teleworkers with TTH (*ps* > 0.062) (see Figure 3).

### Association between coping strategies and headache frequency, and intensity in teleworkers with migraine or TTH

Associations between coping strategies and headache frequency and intensity in teleworkers are presented in Table 3. Regarding migraine profile, results showed that only the use of the denial coping strategy was associated with higher migraine frequency [OR = 1.981, 95% CI (1.222–3.209), *R*^2^ = 0.085, *p* = 0.006] while planning [OR = 0.790, 95% CI (0.627–0.996), *R*^2^ = 0.017, *p* = 0.046] and positive reframing [OR = 0.759, 95% CI (0.597–0.965), *R*^2^ = 0.022, *p* = 0.025] coping strategies were associated with lower migraine intensity. Regarding TTH, results showed that venting [OR = 1.408, 95% CI (1.099–1.805), *R*^2^ = 0.028, *p* = 0.007], self-blame [OR = 1.446, 95% CI (1.142–1.831), *R*^2^ = 0.036, *p* = 0.002] and behavioral disengagement [OR = 1.491, 95% CI (1.141–1.947), *R*^2^ = 0.033, *p* = 0.003] were associated with higher TTH frequency. Results also showed that higher TTH intensity was associated with substance use [OR = 1.284, 95% CI (1.025–1.609), *R*^2^ = 0.015, *p* = 0.030] and behavioral disengagement [OR = 1.495, 95% CI (1.176–1.900), *R*^2^ = 0.035, *p* = 0.001]. As our study population was mostly composed of females and that the mean age of the participants was in the peak prevalence of both headache types, these factors were finally not included as covariate in the regression models. When adjusted for BMI, all previously significant associations remained statistically significant. However, the regression models were not significantly improved by the addition of the BMI covariate, and no significant association were reported between BMI and the headache-related clinical features for either migraine or TTH profile. For this reason, the regression models presented do not include BMI.

**Table 3:** Associations between coping strategies and headache frequency, and intensity in teleworkers.

	Migraine profile				TTH profile			
	Frequency		Intensity		Frequency		Intensity	
	Odds ratio [95%CI]	Value of *p*	Odds ratio [95%CI]	Value of *p*	Odds ratio [95%CI]	Value of *p*	Odds ratio [95%CI]	Value of *p*
Active coping	1.229 [0.847–1.782]	0.278	0.837 [0.663–1.056]	0.134	0.987 [0.788–1.235]	0.909	1.099 [0.893–1.353]	0.373
Planning	1.175 [0.816–1.693]	0.387	0.790 [0.627–0.996]	0.046	1.029 [0.835–1.268]	0.786	1.077 [0.892–1.300]	0.440
Use of instrumental support	1.301 [0.900–1.880]	0.161	0.929 [0.736–1.174]	0.539	1.204 [0.971–1.493]	0.091	1.098 [0.900–1.339]	0.357
Use of emotional support	0.972 [0.658–1.436]	0.886	0.946 [0.748–1.197]	0.645	1.220 [0.978–1.522]	0.078	1.085 [0.886–1.330]	0.429
Venting	1.194 [0.798–1.788]	0.388	0.994 [0.778–1.270]	0.961	1.408 [1.099–1.805]	0.007	1.168 [0.932–1.463]	0.176
Positive reframing	1.019 [0.700–1.485]	0.921	0.759 [0.597–0.965]	0.025	1.042 [0.835–1.300]	0.718	0.905 [0.735–1.114]	0.347
Acceptance	0.796 [0.536–1.183]	0.259	0.920 [0.720–1.175]	0.503	1.056 [0.851–1.310]	0.620	0.825 [0.673–1.011]	0.064
Denial	1.981 [1.222–3.209]	0.006	1.108 [0.778–1.577]	0.570	1.147 [0.821–1.602]	0.421	1.330 [0.980–1.806]	0.067
Self-blame	1.061 [0.736–1.531]	0.750	1.063 [0.848–1.332]	0.596	1.446 [1.142–1.831]	0.002	1.210 [0.977–1.497]	0.080
Humor	0.731 [0.467–1.144]	0.171	0.830 [0.645–1.067]	0.145	0.967 [0.772–1.211]	0.772	0.966 [0.787–1.185]	0.740
Religion	1.253 [0.750–2.092]	0.389	1.142 [0.798–1.635]	0.467	1.156 [0.850–1.572]	0.355	1.199 [0.897–1.603]	0.220
Self-distraction	1.152 [0.779–1.704]	0.478	0.888 [0.701–1.124]	0.323	1.036 [0.800–1.343]	0.788	0.952 [0.746–1.215]	0.691
Substance use	0.862 [0.530–1.402]	0.549	0.927 [0.721–1.192]	0.556	1.119 [0.871–1.437]	0.379	1.284 [1.025–1.609]	0.030
Behavioral disengagement	1.237 [0.806–1.895]	0.331	1.274 [0.958–1.695]	0.095	1.491 [1.141–1.947]	0.003	1.495 [1.176–1.900]	0.001

## Discussion

The objectives of the present study were (1) to describe and compare the headache-related clinical features of teleworkers with primary headache and (2) to determine the association between coping strategies and headache frequency, and intensity in the context of the COVID-19 pandemic. This study included 281 participants (124 with migraine and 157 with TTH). Less than half of participants reported experiencing headache episodes only since the beginning of the pandemic. When looking at headache-related clinical features, teleworkers with migraine reported longer and more painful headache episodes than teleworkers with TTH. However, both groups of teleworkers reported a deterioration of their headache status as well as a decline in their capacity to perform activities of daily living.

Regarding the use of coping strategies, teleworkers with migraine reported using a greater number of coping strategies per individual compared to teleworkers with TTH. The utilization rate of 13 out of the 14 proposed coping strategies was similar between teleworkers with migraine and those with TTH. However, the acceptance coping strategy was used more often in teleworkers with migraine than in teleworkers with TTH. Headache frequency and intensity were both associated with different coping strategies, but the coping strategies associated with headache-related clinical features were different between teleworkers with migraine and those with TTH.

### Sociodemographic data and information related to the working status

The teleworkers’ sociodemographic characteristics were similar between both primary headache profiles. Participants in both groups were mainly middle-aged adults, female (93%) and more than 50% of participants included were considered overweight (BMI ≥ 25 kg/m^2^). BMI has been described in recent systematic reviews as a contributing factor of migraine chronification (39, 40). This increased risk vary between 40 to 80% based on BMI categories (example: normal to obese) (40). For TTH, the implication of BMI in the development and chronification remains unclear (40, 41). In the present study, all associations between coping strategies and headache-related clinical features remain significant when adjusting for BMI and the addition of BMI did not significantly improve the regression models. In addition, there was no significant association between BMI and headache-related clinical features in teleworkers with either migraine or TTH. When looking at the other sociodemographic characteristics, our results are in line with previous studies that reported that both migraine and TTH are more prevalent in women than in men and in middle-aged adults (42–44). Previous studies reported that being female and being aged between 20 and 40 years old were non-modifiable risk factors of developing migraine or TTH (16, 45). In adulthood, the male:female ratio is 0.7 for migraine and 0.8 for TTH (46, 47). The present study is therefore representative of the general primary headache population with regard to age and gender. Further studies that include other socioeconomic data such as family income and level of education as well as comorbidities as potential covariates are needed to better understand the role that these socioeconomic variables could play in the association between coping strategies and headache-related clinical features.

Teleworkers from both primary headache profiles did not differ regarding information related to their work status including, the mean working hours per week, the mean telework hours per week as well as the proportion of teleworkers who had access to a designated office space at home. Participants included in this study are representative of the telework population as at least 90% of their work time is completed in a teleworking situation and as 85% of participants reported having a designated office space at home.

### Headache clinical features

Participants with both migraine and TTH reported similar headache frequency as well as a deterioration of their overall headache status. Even if both migraine and TTH groups reported a deterioration of their headache status, the proportion of teleworkers that had already experienced headache episodes before the pandemic was quite different between the two groups. In fact, about 45% of teleworkers from the TTH profile group have been suffering from headache episodes only since the beginning of the pandemic compared to 22% of teleworkers with migraine. Moreover, teleworkers with migraine reported having more severe and longer headache episodes than teleworkers with TTH as well as using more often medication to treat their headache episodes than teleworkers with TTH. In previous studies, being a woman and having 3 or more headache episodes per month were identified as factors contributing to migraine chronification (48–50). In addition, medication overuse has been reported as an important factor associated with a higher risk of chronification in both migraine and TTH conditions. Overall, these findings support the hypothesis that our group of teleworkers with migraine may be at higher risk of chronification (51, 52). In the present study, teleworkers from both primary headache profiles reported a deterioration of headache status during the pandemic as well as a negative change in capacity to perform activities of daily living. However, headache-related limitations in performing activities of daily living were more frequent in teleworkers with migraine than those with TTH. In migraine as well as in TTH, both headache frequency and intensity are well known to be positively associated with headache-related disability (17, 53, 54). As headache-related disability can represent a substantial economic burden and can be a source of productivity loss due to absenteeism and presenteeism, it is important to better understand what factors affect headache frequency and intensity in teleworkers with headaches (55, 56).

Overall, the current study showed that teleworkers from both primary headache profiles reported a deterioration in headache status characterized by higher headache intensity and episode duration as well as a higher use of medication in teleworkers with migraine. Interestingly, teleworkers with migraine had longer mean headache episodes than teleworkers with TTH, the opposite of what is usually described in the general population. In fact, TTH episodes typically have a duration of 30 min to 7 days while migraine episodes last from 4 to 72 h (36). This difference could be explained by the fact that most teleworkers with migraine already had migraine episodes before the beginning of the pandemic when compared to teleworkers with TTH. Teleworkers with TTH episodes may have had new and shorter headache episodes prompted by their new teleworking conditions. In addition, even if teleworkers from both primary headache profiles reported similar decreased capacity to perform activities of daily living, headache-related limitations were more frequent in teleworkers with migraine. Several behavioral changes were observed in people’s daily living during the pandemic. In people with migraine, social distancing has led to several negative impacts such as a reduction of physical activity, a deterioration of sleep quality, some modifications in food consumption as well as an increase of the time spent at a computer (57).

### Coping strategies

In both groups of primary headache profiles, at least 2 coping strategies were used by each participant to face the COVID-19 pandemic. When looking at the comparison between primary headache profiles regarding the use of coping strategies to face the COVID-19 pandemic, our results showed that all coping strategies were used with similar frequency in both groups. The acceptance coping strategy tends to be slightly more often used by teleworkers with migraine than those with TTH, but this difference was statistically nonsignificant. The large use of acceptance strategy was not surprising in teleworkers with migraine as its use was previously found to be associated with lower migraine disability (58, 59). However, the effectiveness of coping strategies depends on individual preferences, the nature of migraines, and the specific circumstances surrounding them (60, 61). Interestingly, our results suggest that the acceptance coping strategy used to face a new stressful situation, such as the pandemic, did not improve headache symptoms in teleworkers. In other stressful context, the type of coping strategies can be different for each individual and influenced by other factors such as the duration of the stressful context, past experiences and resources (e.g., social support) (62, 63). Regarding the similar use of coping strategies between teleworkers with migraine and those with TTH, a recent review reported that both primary headaches are negatively affected by psychological factors such as stress (64). As the pandemic was a common stressful situation for all participants, it is then not surprising that teleworkers with either migraine or TTH tried to cope using similar coping strategies.

Regarding the association between coping strategies and primary headache frequency, and intensity, results showed that mainly maladaptive coping strategies were associated with headache frequency or headache intensity in teleworkers with migraine or TTH. The denial coping strategy was associated with higher migraine frequency while venting, self-blame and behavioral disengagement were associated with higher TTH frequency among teleworkers. In addition, the planning and the positive reframing coping strategies were associated with lower migraine intensity while substance use and behavioral disengagement were associated with higher TTH intensity. When assessing coping strategies, problem-focus strategies, such as planning and positive reframing, normally include efforts or methods to modify the problem while emotion-focus strategies, such as denial, venting, self-blame and behavioral disengagement, include the management of the emotional distress related to the stressful situation (65). Regarding problem-focus coping strategies, most of them are considered as adaptive coping strategies which is the case for the planning coping strategy. Problem-focused coping strategy have been found to be beneficial when participants are able to take a step back from the problem and are able to take action to correct the problem or situation they are facing (66). In the context of the COVID-19 pandemic, people were able to make some decisions about their family routine, work and lifestyle (67–69). In addition, problem-focused including planning have been previously found to maintain the wellbeing of individuals even during the pandemic (70–72). When looking at emotion-focus strategies, they can be further divided into two subcategories of coping: emotional avoidance coping and emotional approach coping. Emotional avoidance coping styles, including venting, denial, self-blame and behavioral disengagement are considered maladaptive coping strategies while emotional approach coping styles such as acceptance and positive reframing are considered adaptive coping strategies. All emotion-focus strategies have previously been identified as helpful when facing an acute external stressor, but they have also been considered predictors of poorer physical and psychological outcomes in the long term (73–75). Furthermore, they have been associated with a lower adaptation capacity especially in chronic pain context (76–78). The coping strategy positive reframing has been shown to result in more positive psychological adjustment, especially when the stressful situation is uncontrollable (76). This could then explain why positive reframing has been identified as a potential protective coping strategy. By being more rational and less stressed on a daily basis, people may be able to indirectly prevent higher migraine intensity episodes (79).

Overall, coping strategies associated with headache frequency and intensity are different among teleworkers with migraine and those with TTH. Further studies assessing coping strategies over time in teleworkers with primary headache are needed to evaluate the evolution of headache frequency and intensity as home-based telework remains and will remain popular for the years to come.

### Study strengths and limitations

This study has been conducted during the second year of the COVID-19 pandemic in Canada, when teleworking had been implemented for little over a year and teleworkers were no more in an acute adaptation phase of working environment and furniture, but teleworking was still recommended by the government. This is the first study to assess coping strategies in the context of the COVID-19 pandemic in teleworkers suffering from migraine or TTH. Strengths of this study include the large sample size and the recruitment methods that allowed us to include participants from both large cities and remote areas. It is, however, not without limitations. First, it was not possible to compare the results of non-completers with those of completers as the majority of excluded responders did not reach the coping section of the survey. Non-completers may have used different coping strategies than completers which could have led to a non-response bias (80, 81). Second, headache types were determined using only items related to the IHS classification for migraine and tension-type. The questionnaire was self-administered, and it was not possible to further assess the participants’ headache complaint. We therefore had to deduct the diagnosis based on the participants’ answers. Neurological examinations and imaging were not performed due the contact restrictions during the pandemic, and we cannot exclude that some participants had an underlying medical condition causing headache. However, all participants for which items related to symptoms and the general profile of headache were not concordant were excluded. Third, headache frequency and intensity were self-reported by participants based on the previous month and year which may have led participants to report only the most important headache episodes they could remembered. Fourth, as this study was conducted using a cross-sectional design, it was not possible to capture changes that might influence participants’ symptoms, such as medication, weight, anxiety, sleeping quality (57) or changes related to the use of coping strategies. Fifth, because migraine and TTH are the most prevalent primary headaches, the present study only focused on these two. Therefore, the current results cannot be generalized to all primary headache types nor to secondary headache types. Finally, the context related to the COVID-19 surrounding the government policies may have been different in Quebec than in other Canadian provinces. For this reason, results cannot be generalized outside of Quebec borders.

### Clinical implications

Results of the present study show the role of coping strategies in managing stressful situations that impact headache symptoms. Therefore, the assessment of coping strategies offers a unique opportunity for clinicians to provide support and guidance to individuals with headache. In addition to coping strategy assessment, clinicians can provide teleworkers with specific advice regarding optimization of the home workspace and strategies to minimize environmental triggers, such as taking regular breaks and managing noise.

## Conclusion

The results of the present study showed that teleworkers with migraine and TTH had similar sociodemographic profiles and work demands. However, a higher proportion of teleworkers with migraine already experienced headache episodes before the beginning of the pandemic compared to teleworkers with TTH. Teleworkers with migraine had more painful and longer headache episodes, experienced more frequent headache-related limitations and used medication more often than teleworkers with TTH. Regarding coping strategies, even if teleworkers with both primary headache profiles reported a similar frequency of use for most coping strategies, the acceptance strategy was used more frequently by teleworkers with migraine compared to those with TTH. Finally, coping strategies that have been found to be associated with headache frequency and intensity were quite different among teleworkers with migraine and those with TTH. Most coping strategies were maladaptive, except the planning and the positive reframing coping strategies that were found to be inversely associated with migraine intensity. Further studies including follow-up should be conducted to be able to determine if the use of such coping strategies remain associated with headache-related clinical features over time and, if yes, how they impact these features in the long term.

## Data availability statement

The original contributions presented in the study are included in the article/supplementary material, further inquiries can be directed to the corresponding author.

## Ethics statement

The studies involving humans were approved by the Human Research Ethics Board of “Université du Québec à Trois-Rivières” (CER-21-277-07.18) and all methods were performed in accordance with the relevant guidelines and regulations. All participants provided informed written consent before completing the online survey.

## Author contributions

MH: Conceptualization, Formal analysis, Investigation, Methodology, Writing – original draft, Writing – review & editing. JD: Writing – review & editing, Conceptualization, Formal analysis, Investigation, Methodology, Writing – original draft. AL: Conceptualization, Formal analysis. MD: Writing – review & editing, Conceptualization, Methodology, Project administration, Supervision. A-AM: Conceptualization, Methodology, Supervision, Writing – original draft. JA: Writing – original draft, Writing – review & editing, Conceptualization, Investigation, Methodology, Project administration, Resources, Supervision.

## References

[1] Donthu N Gustafsson A. Effects of COVID-19 on business and research. J Bus Res. (2020) 117:284–289. doi: 10.1016/j.jbusres.2020.06.008 32536736 PMC7280091

[2] Kaushik M Guleria N. The impact of pandemic COVID-19 in workplace. Eur J Busi Manag. (2020) 12:1–10. doi: 10.7176/EJBM/12-15-02

[3] Cahapay MB. Social distancing practices of residents in a Philippine region with low risk of COVID-19 infection. Eur J Environ and Public Health. (2020):4. doi: 10.29333/ejeph/8455

[4] Buomprisco G Ricci S Perri R de Sio S. Health and telework: new challenges after COVID-19 pandemic. Eur J Environ and Public Health. (2021) 5:em0073. doi: 10.21601/ejeph/9705

[5] Pulido-Martos M Cortés-Denia D Lopez-Zafra E. Teleworking in times of COVID-19: effects on the acquisition of personal resources. Front Psychol. (2021) 12:2485. doi: 10.3389/fpsyg.2021.685275 PMC8262645 34248789

[6] McGrail DJ Dai J McAndrews KM Kalluri R. Enacting national social distancing policies corresponds with dramatic reduction in COVID19 infection rates. PLoS One. (2020) 15:e0236619. doi: 10.1371/journal.pone.0236619 32730356 PMC7392246

[7] Olney AM Smith J Sen S Thomas F Unwin HJT. Estimating the effect of social distancing interventions on COVID-19 in the United States. Am J Epidemiol. (2021) 190:1504–9. doi: 10.1093/aje/kwaa293, PMID: 33406533 PMC7929448

[8] Mofijur M Fattah IR Alam MA Islam ABMS Ong HC Rahman SMA. Impact of COVID-19 on the social, economic, environmental and energy domains: lessons learnt from a global pandemic. Sustain Prod Consumpt. (2021) 26:343–59. doi: 10.1016/j.spc.2020.10.016, PMID: 33072833 PMC7556229

[9] Belzunegui-Eraso A Erro-Garcés A. Teleworking in the context of the Covid-19 crisis. Sust. (2020) 12:3662. doi: 10.3390/su12093662, PMID: 36467168

[10] Allen TD Golden TD Shockley KM. How effective is telecommuting? Assessing the status of our scientific findings. Psychol Sci Public Interest. (2015) 16:40–68. doi: 10.1177/1529100615593273, PMID: 26403188

[11] Messenger JC Gschwind L. Three generations of telework: new ICT s and the (R) evolution from Home Office to virtual office. N Technol Work Employ. (2016) 31:195–208. doi: 10.1111/ntwe.12073

[12] Morgan RE. Teleworking: an assessment of the benefits and challenges. Eur Bus Rev. (2004) 16:344–57. doi: 10.1108/09555340410699613, PMID: 34865097

[13] Kniffin KM Narayanan J Anseel F Antonakis J Ashford SP Bakker AB. COVID-19 and the workplace: implications, issues, and insights for future research and action. Am Psychol. (2021) 76:63–77. doi: 10.1037/amp0000716 32772537

[14] Mehdi T René M. Working from home: productivity and preferences. StatCan COVID-19: data to insights for a better Canada. Statistics Canada. Catalogue. (2021). doi: 10.25318/36280001202100500001-eng

[15] Spencer LJ Degu A Kalkidan HA Solomon MA Cristiana A Nooshin A. Global, regional, and national incidence, prevalence, and years lived with disability for 354 diseases and injuries for 195 countries and territories, 1990-2017: a systematic analysis for the global burden of disease study 2017. Lancet. (2018) 392:1789–858. doi: 10.1016/s0140-6736(18)32279-7 30496104 PMC6227754

[16] Stovner LJ Hagen K Linde M Steiner TJ. The global prevalence of headache: an update, with analysis of the influences of methodological factors on prevalence estimates. J Headache Pain. (2022) 23:20220412. doi: 10.1186/s10194-022-01402-2 PMC9004186 35410119

[17] Houle M Lessard A Marineau-Bélanger É Lardon A Marchand AA Descarreaux M. Factors associated with headache and neck pain among telecommuters–a five days follow-up. BMC Public Health. (2021) 21:1–10. 34090415 10.1186/s12889-021-11144-6 PMC8179834

[18] Nash JM Thebarge RW. Understanding psychological stress, its biological processes, and impact on primary headache. Headache. (2006) 46:1377–86. doi: 10.1111/j.1526-4610.2006.00580.x 17040334

[19] Ashina S Lyngberg A Jensen R. Headache characteristics and chronification of migraine and tension-type headache: a population-based study. Cephalalgia. (2010) 30:943–54. doi: 10.1177/0333102409357958, PMID: 20656705

[20] Garfin DR. Technology as a coping tool during the COVID-19 pandemic: implications and recommendations. Stress Health. (2020) 36:555–9. doi: 10.1002/smi.2975, PMID: 32762116 PMC7436915

[21] Hirschberger G. Collective trauma and the social construction of meaning. Front Psychol. (2018) 9:1441. doi: 10.3389/fpsyg.2018.01441 30147669 PMC6095989

[22] Ogueji IA Okoloba MM Demoko Ceccaldi BM. Coping strategies of individuals in the United Kingdom during the COVID-19 pandemic. Curr Psychol. (2021) 41:7493–9. doi: 10.1007/s12144-020-01318-7 33424202 PMC7779093

[23] Monteiro AMF Santos RL Kimura N Baptista MAT Dourado MCN. Coping strategies among caregivers of people with Alzheimer disease: a systematic review. Trends Psychiatry Psychother. (2018) 40:258–68. doi: 10.1590/2237-6089-2017-0065, PMID: 30304119

[24] Snyder CR. Coping: the psychology of what works. USA: Oxford University Press (1999).

[25] Carver CS Scheier MF. Situational coping and coping dispositions in a stressful transaction. J Pers Soc Psychol. (1994) 66:184. doi: 10.1037/0022-3514.66.1.184, PMID: 8126648

[26] Lazarus RS Folkman S. Stress, appraisal, and coping. New York, NY: Springer publishing company (1984).

[27] Carver CS Scheier MF Weintraub JK. Assessing coping strategies: a theoretically based approach. J Pers Soc Psychol. (1989) 56:267–83. doi: 10.1037/0022-3514.56.2.267, PMID: 2926629

[28] Matsuzawa Y Lee YSC Fraser F Langenbahn D Shallcross A Powers S. Barriers to behavioral treatment adherence for headache: an examination of attitudes, beliefs, and psychiatric factors. Headache: the journal of head and face. Pain. (2019) 59:19–31. doi: 10.1111/head.13429 PMC6344047 30367821

[29] Keefe FJ Rumble ME Scipio CD Giordano LA Perri LCM. Psychological aspects of persistent pain: current state of the science. J Pain. (2004) 5:195–211. doi: 10.1016/j.jpain.2004.02.576, PMID: 15162342

[30] Marjanovic Z Greenglass ER Coffey S. The relevance of psychosocial variables and working conditions in predicting nurses’ coping strategies during the SARS crisis: an online questionnaire survey. Int J Nurs Stud. (2007) 44:991–8. doi: 10.1016/j.ijnurstu.2006.02.012, PMID: 16618485 PMC7094220

[31] Sim K Huak Chan Y Chong PN Chua HC Wen Soon S. Psychosocial and coping responses within the community health care setting towards a national outbreak of an infectious disease. J Psychosom Res. (2010) 68:195–202. doi: 10.1016/j.jpsychores.2009.04.004, PMID: 20105703 PMC7094450

[32] Smitherman TA Ward TN. Psychosocial factors of relevance to sex and gender studies in headache. Headache. (2011) 51:923–31. doi: 10.1111/j.1526-4610.2011.01919.x, PMID: 21631477

[33] Hawker GA Mian S Kendzerska T French M. Measures of adult pain: visual analog scale for pain (vas pain), numeric rating scale for pain (nrs pain), mcgill pain questionnaire (mpq), short-form mcgill pain questionnaire (sf-mpq), chronic pain grade scale (cpgs), short form-36 bodily pain scale (sf-36 bps), and measure of intermittent and constant osteoarthritis pain (icoap). Arthritis Care Res. (2011) 63:S240–52. doi: 10.1002/acr.20543 22588748

[34] Muller L Spitz E. Multidimensional assessment of coping: validation of the brief COPE among French population. L’Encéphale. (2003) 29:507–18. PMID: 15029085

[35] Carver CS. You want to measure coping but your protocol’too long: consider the brief cope. Int J Behav Med. (1997) 4:92–100. doi: 10.1207/s15327558ijbm0401_6, PMID: 16250744

[36] Society HCCotIH. The international classification of headache disorders, (beta version). Cephalalgia. (2013) 33:629–808. doi: 10.1177/0333102413485658, PMID: 23771276

[37] Boonstra AM Stewart RE Köke AJ Oosterwijk RFA Swaan JL Schreurs KMG. Cut-off points for mild, moderate, and severe pain on the numeric rating scale for pain in patients with chronic musculoskeletal pain: variability and influence of sex and catastrophizing. Front Psychol. (2016) 7:1466. doi: 10.3389/fpsyg.2016.01466, PMID: 27746750 PMC5043012

[38] Knox EM Ng RT. Algorithms for mining distancebased outliers in large datasets In: Proceedings of the international conference on very large data bases. New York, USA: Citeseer (1998). 392–403.

[39] Ornello R Ripa P Pistoia F Degan D Tiseo C Carolei A. Migraine and body mass index categories: a systematic review and meta-analysis of observational studies. J Headache Pain. (2015) 16:1–14. doi: 10.1186/s10194-015-0510-z 25903159 PMC4385329

[40] Chai NC Scher AI Moghekar A Bond DS Peterlin BL. Obesity and headache: part I--a systematic review of the epidemiology of obesity and headache. Headache. (2014) 54:219–34. doi: 10.1111/head.12296, PMID: 24512574 PMC3971380

[41] Bigal ME Tsang A Loder E Serrano D Reed ML Lipton RB. Body mass index and episodic headaches: a population-based study. Arch Intern Med. (2007) 167:1964–70. doi: 10.1001/archinte.167.18.1964, PMID: 17923596

[42] Allais G Chiarle G Sinigaglia S Airola G Schiapparelli P Benedetto C. Gender-related differences in migraine. Neurol Sci. (2020) 41:429–36. doi: 10.1007/s10072-020-04643-8, PMID: 32845494 PMC7704513

[43] Fuensalida-Novo S Parás-Bravo P Jiménez-Antona C Castaldo M Wang K Benito-González E. Gender differences in clinical and psychological variables associated with the burden of headache in tension-type headache. Women Health. (2020) 60:652–63. doi: 10.1080/03630242.2019.1696440, PMID: 31795922

[44] al-Hassany L Haas J Piccininni M Kurth T Maassen van den Brink A Rohmann JL. Giving researchers a headache–sex and gender differences in migraine. Front Neurol. (2020) 11:549038. doi: 10.3389/fneur.2020.549038, PMID: 33192977 PMC7642465

[45] Vetvik KG MacGregor EA. Sex differences in the epidemiology, clinical features, and pathophysiology of migraine. Lancet Neurol. (2017) 16:76–87. doi: 10.1016/s1474-4422(16)30293-9 27836433

[46] Lyngberg AC Rasmussen BK Jørgensen T Jensen R. Prognosis of migraine and tension-type headache: a population-based follow-up study. Neurology. (2005) 65:580–5. doi: 10.1212/01.wnl.0000172918.74999.8a 16116119

[47] Jensen R Stovner LJ. Epidemiology and comorbidity of headache. Lancet Neurol. (2008) 7:354–61. doi: 10.1016/S1474-4422(08)70062-0, PMID: 18339350

[48] Finocchi C Strada L. Sex-related differences in migraine. Neurol Sci. (2014) 35:207–13. doi: 10.1007/s10072-014-1772-y, PMID: 24867868

[49] Lipton RB Stewart WF Diamond S Diamond ML Reed M. Prevalence and burden of migraine in the United States: data from the American migraine study II. Headache: the journal of head and face. Pain. (2001) 41:646–57. doi: 10.1046/j.1526-4610.2001.041007646.x 11554952

[50] Scher A Stewart W Ricci J Lipton BR. Factors associated with the onset and remission of chronic daily headache in a population-based study. Pain. (2003) 106:81–9. doi: 10.1016/S0304-3959(03)00293-8, PMID: 14581114

[51] Cevoli S Sancisi E Grimaldi D Pierangeli G Zanigni S Nicodemo M. Family history for chronic headache and drug overuse as a risk factor for headache Chronification. J Head Face Pain. (2009) 49:412–8. doi: 10.1111/j.1526-4610.2008.01257.x 19267785

[52] May A Schulte LH. Chronic migraine: risk factors, mechanisms and treatment. Nat Rev Neurol. (2016) 12:455–64. doi: 10.1038/nrneurol.2016.93, PMID: 27389092

[53] Magnusson JE Becker WJ. Migraine frequency and intensity: relationship with disability and psychological factors. Headache. (2003) 43:1049–59. doi: 10.1046/j.1526-4610.2003.03206.x, PMID: 14629240

[54] Stewart WF Lipton RB Kolodner K. Migraine disability assessment (MIDAS) score: relation to headache frequency, pain intensity, and headache symptoms. Headache: the journal of head and face. Pain. (2003) 43:258–65. doi: 10.1046/j.1526-4610.2003.03050.x 12603645

[55] Begasse de Dhaem O Gharedaghi MH Bain P Hettie G Loder E Burch R. Identification of work accommodations and interventions associated with work productivity in adults with migraine: a scoping review. Cephalalgia. (2021) 41:760–73. doi: 10.1177/0333102420977852, PMID: 33302697

[56] Baigi K Stewart WF. Headache and migraine: a leading cause of absenteeism. Handbook Clin Neurol Elsevier. (2015) 131:447–63. doi: 10.1016/B978-0-444-62627-1.00025-1 26563803

[57] di Stefano V Ornello R Gagliardo A Torrente A Illuminato E Caponnetto V. Social distancing in chronic migraine during the COVID-19 outbreak: results from a multicenter observational study. Nutrients. (2021) 13:1361. doi: 10.3390/nu13041361, PMID: 33921674 PMC8074143

[58] Chan JK Consedine NS. Negative affectivity, emotion regulation, and coping in migraine and probable migraine: a New Zealand case–control study. Int J Behav Med. (2014) 21:851–60. doi: 10.1007/s12529-013-9370-6, PMID: 24242822

[59] Dindo L Recober A Marchman J O’Hara M Turvey C. Depression and disability in migraine: the role of pain acceptance and values-based action. Int J Behav Med. (2015) 22:109–17. doi: 10.1007/s12529-014-9390-x, PMID: 24515397

[60] Thoits PA. Stress, coping, and social support processes: where are we? What next? J Health Soc Behav. (1995):53–79. doi: 10.2307/2626957, PMID: 7560850

[61] Oakland S Ostell A. Measuring coping: a review and critique. Hum Relat. (1996) 49:133–55. doi: 10.1177/001872679604900201, PMID: 37772763

[62] Hudson K. Coping complexity model: coping stressors, coping influencing factors, and coping responses. Psychology. (2016) 7:300. doi: 10.4236/psych.2016.73033

[63] McFadden P Ross J Moriarty J Mallett J Schroder H Ravalier J. The role of coping in the wellbeing and work-related quality of life of UK health and social care workers during COVID-19. Int J Environ Res Public Health. (2021) 18:815. doi: 10.3390/ijerph18020815, PMID: 33477880 PMC7832874

[64] Onan D Younis S Wellsgatnik WD Farham F Andruškevičius S Abashidze A. Debate: differences and similarities between tension-type headache and migraine. J Headache Pain. (2023) 24:92. doi: 10.1186/s10194-023-01614-0 37474899 PMC10360340

[65] Baker JP Berenbaum H. Emotional approach and problem-focused coping: a comparison of potentially adaptive strategies. Cognit Emot. (2007) 21:95–118. doi: 10.1080/02699930600562276

[66] Allen AB Leary MR. Self-compassion, stress, and coping. Soc Personal Psychol Compass. (2010) 4:107–18. doi: 10.1111/j.1751-9004.2009.00246.x, PMID: 20686629 PMC2914331

[67] van der Werf ET Busch M Jong MC Hoenders HJR. Lifestyle changes during the first wave of the COVID-19 pandemic: a cross-sectional survey in the Netherlands. BMC Public Health. (2021) 21:1226. doi: 10.1186/s12889-021-11264-z 34172042 PMC8231077

[68] Suffren S Dubois-Comtois K Lemelin J-P St-Laurent D Milot T. Relations between child and parent fears and changes in family functioning related to COVID-19. Int J Environ Res Public Health. (2021) 18:1786. doi: 10.3390/ijerph18041786, PMID: 33673155 PMC7918466

[69] Vaziri H Casper WJ Wayne JH Matthews RA. Changes to the work–family interface during the COVID-19 pandemic: examining predictors and implications using latent transition analysis. J Appl Psychol. (2020) 105:1073–87. doi: 10.1037/apl0000819, PMID: 32866024

[70] Budimir S Probst T Pieh C. Coping strategies and mental health during COVID-19 lockdown. J Ment Health. (2021) 30:156–63. doi: 10.1080/09638237.2021.1875412, PMID: 33502917

[71] Gurvich C Thomas N Thomas EH Hudaib AR Sood L Fabiatos K. Coping styles and mental health in response to societal changes during the COVID-19 pandemic. Int J Soc Psychiatry. (2021) 67:540–9. doi: 10.1177/0020764020961790, PMID: 33016171

[72] al Kindi RM Ambusaidi AA al Mahrooqi AY al Omrani NH al Jabri MK al Sumri HH. Factors associated with coping strategies due to covid-19 pandemic-related stressors among Omani adults. Oman Med J. (2022) 37:e424. doi: 10.5001/omj.2022.84, PMID: 36188878 PMC9453778

[73] Penley JA Tomaka J Wiebe JS. The association of coping to physical and psychological health outcomes: a meta-analytic review. J Behav Med. (2002) 25:551–603. doi: 10.1023/A:1020641400589, PMID: 12462958

[74] Oryan Z Avinir A Levy S Kodesh E Elkana O. Risk and protective factors for psychological distress during COVID-19 in Israel. Curr Psychol. (2021) 42:2448–59. doi: 10.1007/s12144-021-02031-9 34248314 PMC8257084

[75] Fluharty M Bu F Steptoe A Fancourt D. Coping strategies and mental health trajectories during the first 21 weeks of COVID-19 lockdown in the United Kingdom. Soc Sci Med. (2021) 279:113958. doi: 10.1016/j.socscimed.2021.113958, PMID: 33965772 PMC9756769

[76] Ewert C Vater A Schröder-Abé M. Self-compassion and coping: a meta-analysis. Mindfulness. (2021) 12:1063–77. doi: 10.1007/s12671-020-01563-8, PMID: 37197599

[77] Connor-Smith JK Flachsbart C. Relations between personality and coping: a meta-analysis. J Pers Soc Psychol. (2007) 93:1080. doi: 10.1037/0022-3514.93.6.1080, PMID: 18072856

[78] Dysvik E Natvig GK Eikeland O-J Lindstrøm TC. Coping with chronic pain. Int J Nurs Stud. (2005) 42:297–305. doi: 10.1016/j.ijnurstu.2004.06.009, PMID: 15708016

[79] Sauro KM Becker WJ. The stress and migraine interaction. Headache. (2009) 49:1378–86. doi: 10.1111/j.1526-4610.2009.01486.x, PMID: 19619238

[80] Berg N. Non-response bias. In: Kempf-Leonard K. (Editor) Encyclopedia of Social Measurement, Elsevier (2005) 865–873.

[81] Hill A Roberts J Ewings P Gunnell D. Non-response bias in a lifestyle survey. J Public Health. (1997) 19:203–7. doi: 10.1093/oxfordjournals.pubmed.a024610 9243437

